# The Thomas More Lists: A Phonemically Balanced Dutch Monosyllabic Speech Audiometry Test

**DOI:** 10.3390/audiolres12040041

**Published:** 2022-07-29

**Authors:** Filiep Vanpoucke, Marleen De Sloovere, Anke Plasmans

**Affiliations:** 1 Department of Speech & Language Therapy and Audiology, Thomas More University of Applied Sciences, 2000 Antwerp, Belgium; marleen.desloovere@thomasmore.be; 2 Cochlear Technology Centre, 2800 Mechelen, Belgium; aplasmans@cochlear.com

**Keywords:** speech audiometry, phonemic balance, speech recognition curve

## Abstract

Speech audiometry tests are a crucial tool in clinical care and research. In Dutch, the common practice is to use lists of monosyllabic words with a consonant-vowel-consonant (CVC) structure. However, there are relatively few lists, and they are short. Here, the goal is to develop an adult speech audiometry test for Dutch (Flemish) consisting of phonemically balanced lists of 25 CVC words. The ISO 8253-3:2012 norm was followed. From a pool of 689 well-known words, an initial set of 26 lists was recorded by a female speaker. The lists were optimized for perceptual balance by means of two studies with young normal hearing listeners (N_1_ = 24, N_2_ = 32). The final corpus contains 16 phonetically and perceptually balanced lists. In a last study (N_3_ = 25), the reference speech recognition curves in quiet and in speech-shaped noise were determined. Reference speech recognition threshold and slope values for phoneme scoring are respectively 20.3 dB_SPL_ in quiet (slope 5.2%/dB) and −7.7 dB_SNR_ (7.5%/dB) in noise, similar to existing materials. The lists may be a useful addition to the existing audiometric tests.

## 1. Introduction

Speech audiometry is a standard clinical procedure used at various stages along the diagnosis and treatment pathway for patients experiencing hearing loss. It is a key measure for characterizing the nature and severity of the hearing loss, evaluating candidacy for various hearing solutions (e.g., hearing aids or implants), validating device fitting, and monitoring long-term performance. As an example, the current Belgian rules for reimbursement of a unilateral cochlear implant (CI) state that the phoneme recognition score on a monosyllabic test at 70 dB_SPL_ in a quiet setting should not exceed 50% (unaided, free field). In addition to clinical applications, studies in the field of hearing research frequently include a speech understanding objective, translated into some speech audiometry endpoint. 

The exact nature and configuration of the speech audiometry test varies depending on the specific purpose. Speech materials can be mono- or polysyllabic words, word sequences, or meaningful sentences. They can be presented in quiet or in some background noise, at fixed or adaptive levels, and at differing sound source locations. Tests with single words in a quiet setting are meant to assess analytical (bottom-up) hearing performance. Such words of short duration often take the form of a CVC (one consonant-one vowel-one consonant) structure. For the Dutch language, CVC is the most common syllable structure. An analysis of a subset of the Corpus of Spoken Dutch containing 855,892 words with a verified phonetic transcription showed that 32% of the syllables had this structure [1]. Tests with longer materials, such as meaningful sentences in noise, are preferred when the goal is assessment of a person’s comprehension under more realistic conditions. An overview of available Dutch speech audiometry materials can be found in Hammer et al. [2]. To measure a person’s speech discrimination ability, the Nederlandse Vereniging Audiologie (NVA) speech audiometry test is commonly used in the Dutch speaking part of Belgium, Flanders. The NVA test was originally developed in 1992 by the Dutch Audiology Association in the Netherlands [3]. As pronunciation differences exist between the Netherlands and Flanders, a Flemish version of the NVA word materials was recorded and normed [4].

Although widely used, the Dutch NVA test has some limitations. A first issue is the small number of available lists. Only 15 lists each containing 12 words are available, forming a pool of 177 CVC words (three words are repeated). This number is rather small, given its wide use in clinical care and research. A speech corpus with a larger set of materials would avoid re-use of the same words and lists, which is particularly important for research, which often requires comparing several conditions [5].

Secondly, the number of test items affects both the sensitivity and variability of a speech test. In the NVA test, the first word of each list is a practice item and is not counted, leaving only 11 words to be scored per list. Scoring is performed at the phoneme level (33 phonemes), and therefore a single phoneme error (a substitution, deletion, or insertion) represents 3% of the final score for that list. Small but significant differences in hearing performance may go unnoticed. Test-retest variability is also influenced by the number of test items, as the variability on a measured speech score decreases with the square root of the number of test items [6,7,8]. Although the NVA lists contain 33 phonemes, some predictability exists because not every random combination of a consonant, vowel, and consonant constitutes a valid and familiar Dutch word. Boothroyd & Nittrouer found for CVC words an empirical value of 2.5 phonemes per word, leading to an effective N of ±27.5 phonemes, when applied to a NVA test list [9]. Around speech recognition threshold, the standard error of the mean is then rather high, approximately 9.5%. To increase the number of test items, a pragmatic solution could be to use more lists for every experimental condition, but this is limited by the small number of available lists. 

Thirdly, the NVA test is not phonemically balanced. Its phoneme distribution does not match the distribution found in daily speech [2]. The phoneme sets for the initial, middle, and final position contain respectively 16 consonants, 12 vowels, and 12 consonants. Given there are only 11 test words in each list, a phoneme typically occurs once per list. The phoneme distribution, averaged over the 15 test lists, is indicated with the blue bars in Figure 2. The distribution is rather uniform, deviating strongly from the Dutch language statistics. As an example, the common /r/ sound is even absent. The importance of phonemic balancing is debated. In general, research studies on this topic tend to conclude that phonemic balancing is not the most important criterion [10], but it makes the word lists more valid [2,11,12,13].

A final minor point is that the frequency or familiarity of the NVA words is not documented. It is well known that when all other factors are equal, more frequently occurring words have higher intelligibility [14,15].

The purpose of the currently presented work is to develop a new Dutch speech audiometry test consisting of monosyllabic words with a consonant-vowel-consonant structure with longer, more phonemically balanced lists. Longer lists come with a risk of increased listening effort, possibly resulting in a negative influence on the test result. Therefore, a list length of 25 words was selected, already resulting in a reduction of the standard deviation by a factor of 1.51.

The ISO 8253-3:2012 standard “Acoustics—Audiometric test methods—Part 3: Speech audiometry” (International Organization for Standardization, Geneva, Switzerland) was used to guide the development. 

## 2. Materials and Methods

### 2.1. Development of the Audio Materials

As it was the intention to create many lists, it was important to maximize the size of the CVC word pool (lexicon) to select from. A computer program listed all combinations of a set of 18 leading consonants (/b, d, f, h, j, k, l, m, n, p, r, s, t, v, w, z, Ɣ, ʃ/), 15 medial vowels and diphthongs (/a:, e:, i, o:, u, y, ø, œy, ɑ, ɑu, ɔ, ɛ, ɛi, ɪ, ʏ/), and 13 final consonants (/f, j, k, l, m, n, p, r, s, t, w, x, ŋ/). The candidate triphone words were spelled out. In a first step, two native speakers selected from the 3510 written monosyllables the existing Dutch words, resulting in a pool of 1041 nouns, verbs, pronouns, adverbs, and adjectives. No restrictions were imposed based on lexical category. Inappropriate words were removed. Finally, the pool was filtered for word familiarity using a recent data set from a large population survey (±400,000 native speakers of Flemish and Dutch, age 12 years and above) [16]. Only words that were known by more than 90% of the population were selected. The resulting lexicon contained 689 CVC words. 

Throughout the project, custom programs were created in a mathematical scripting language (Matlab R2017a, MathWorks, Natick, MA, USA) to implement all processing steps. A first step was to compose phonemically balanced lists by selecting words from the lexicon. A phonetic dictionary was created containing the international phonetic alphabet (IPA) transcription for each word. The transcriptions were validated by three native speakers. Averaging over this dictionary, the relative frequency of each phoneme in each position was determined. This lexicon distribution served as the target phonemic distribution, driving the word selection process for each list. By construction, the lexicon distribution approximates closely the phonemic frequency in the Dutch language for the subset of words satisfying a CVC structure. It still differs from the phonemic distribution in the general language as the Dutch vocabulary contains many polysyllabic and other structures. Additionally, it is a type frequency distribution, reflecting the phoneme distribution in a lexicon, ignoring how often a certain word is actually used in spoken or written language. Token frequencies are more reflective of language use and may result in slight changes to the phonemic distribution [17]. 

For the initial version of the corpus, 26 lists were created. The target phoneme counts for each list in each position were set by multiplying the lexicon phonemic distribution with the list length. As an example, the phoneme /p/ occurred in 43 out of the 689 words in the initial position, yielding a 6.2% type frequency. Therefore, its target count, averaged across lists, is 1.55 occurrences in a list of 25 words. The word selection process was driven by a phoneme allocation table (PAT) [18], keeping track of the cumulative phoneme counts per position. Initially, all PAT counts were set to zero. A custom script randomly selected a first word. Its IPA transcription was added to the PAT. Once a word had been selected, it was removed from the search pool. For selecting the next word, an algorithm determined for each remaining word to what extent its addition would reduce the difference between target and current PAT count. From the best scoring words, a random word was chosen, the PAT was updated, and the word selection process repeated itself until the list was fully populated. At the end of every list a small approximation error remained. With a growing number of lists leading to a shrinking search pool, this error tended to increase slightly. In a final step, every list was shuffled to minimize the phonemic similarity of consecutive words, with the intention to reduce auditory memory effects during speech testing.

A female professional speaker produced all the words twice. Following the ISO standard, the recordings took place in a double-walled soundproof booth using a high-quality microphone with a flat frequency response (studio Projects B1 condenser), connected to an RME UFX sound card. The utterances were digitized with a 16-bit A/D converter at a sampling frequency of 44.1 kHz using version 2.4.2 of the Audacity^®^ recording and editing software [19]. The recordings were further digitally processed by custom Matlab scripts. Low-frequency background noise was removed. The individual word recordings were segmented and trimmed, and their broadband root mean square (RMS) level was adjusted to −25 dB_FS_. Two native speakers assessed the two utterances of each word for articulation, prosody, and noise and selected the best one. 

For each study described below, a CD was produced. The CD creation process started with the production of a mono audio file for each list, in which the words were concatenated with a 4.3 s silence interval separating them. A spectrally matching speech-shaped noise was created by randomly time-shifting and adding all words of all lists together many times until a stationary noise was obtained. The audio was stored in a 16-bit, 44.1 kHz stereo format with clean speech and noise respectively on track 1 and 2. Both tracks were calibrated at −25 dB_FS_.

### 2.2. Design and Validation of the Speech Lists

It is crucial that the performance across lists is matched so that the choice of a particular list has little or no result on the hearing outcome. Perceptual balance implies minimal variance across lists when tested under equivalent conditions. We chose to test perceptual balance at the most sensitive point of the curve, namely the Speech Recognition Threshold level (SRT), defined as the speech level corresponding to a 50% speech recognition score. List SRTs should differ by no more than 1 dB.

To develop the final corpus, three iterations, each requiring data collection with human subjects, were required. The studies were reviewed and approved by the internal scientific advisory board of the Speech-Language and Audiology Department at Thomas More University of Applied Sciences. Prior written informed consent was obtained from all participants. The number of participants varied across the studies. They were young normal-hearing native speakers from Flanders (age 18 to 25 years), mainly female. Otologic normality was checked with otoscopy, tympanometry, and a tonal audiometry test, following the ISO 8253-3:2012 recommendation to define normal hearing as an average tone threshold (octave and mid-octave frequencies ranging from 250 to 8000 Hz included) equal to or better than 10 dB_HL_ and a maximum of two frequencies having a threshold of 15 dB_HL_. 

The speech lists were presented monaurally through a TDH 39 audiometric headset (Telephonics, Farmingdale, NY, USA) in a double-walled soundproof booth. By default, the right ear was tested. If this ear did not meet the inclusion criteria, the left ear was evaluated. Speech audiometry was measured via the OtoSuite software, version 4.84 (Natus Medical, Middleton, San Carlos, CA, USA) on the audiometer PC (Dell Latitude E5440), controlling a Madsen Astera Otometrics audiometer. The audiometric setup was calibrated prior to each experiment. Subjects were instructed to repeat aloud whatever was heard. The subject’s oral responses were registered by an omnidirectional microphone and passed to the headphones of the investigator, sitting outside of the booth. 

The initial version of the corpus containing 26 speech lists was evaluated in a first study in a group of 24 young normal hearing subjects (20 female, 4 male). Each subject listened to all lists in quiet, covering a range of 20–45 dB_SPL_ in steps of 5 dB_SPL_, selected as per a randomization table. In line with the ISO 8253-3:2012 standard, the reference speech recognition curve in quiet was determined as the median speech recognition value across participants. The main purpose of this first phase was to select words with similar speech intelligibility, a step intended to optimize the steepness of the psychometric function. The SRT of each individual word was estimated based on the available data. On average, 4.3 subjects (26 lists spread over 6 levels) tested a particular {word, level} combination. This is a small number still leaving considerable uncertainty on the estimate of the median value. The same word was evaluated by other subjects at different presentation levels. A sigmoid function was fitted to estimate the 50% intelligibility point of each individual word. The ISO 8253-3:2012 norm proposes to filter out words whose SRT value deviated more than 3 dB_SPL_ from the list average. Application of this approach resulted in a loss of too many words. In a first attempt to alleviate this, level corrections were applied to the individual words, compensating for either 50 or 100% of the SRT difference. A small pilot with five normal hearing listeners, evaluating the lists with level corrections, was unconvincing. The level corrections were not very effective in equalizing the word SRTs, and loudness variations were noticeable, certainly with the 100% compensation. Instead, a more relaxed 5 dB_SPL_ criterion was applied, resulting in a reduction of the world pool from 650 to 500 words. 

In the second iteration, from the 500 words, 20 new phonemically balanced lists were composed and a new CD was created. A group of 32 new young normal hearing listeners (17 female, 15 male) listened to all 20 lists spread over a 15–50 dB_SPL_ range (steps of 5 dB). The focus was now on selecting lists satisfying the 1 dB perceptual balance criterion mentioned in the ISO 8253-3:2012 standard. Four lists did not meet the criterion and were removed. The reference speech recognition curve in quiet was determined on the remaining 16 lists.

A speech-shaped noise matching the spectrum of all words in the final 16 lists was generated, and a new CD was made. In the third study with 25 new young normal hearing participants (22 female, 3 male), the reference psychometric curve for speech recognition in noise was determined. Every participant listened to all lists, this time distributed over a range of −16 to 0 dB_SNR_ in steps of 2 dB. The total presentation level of the speech and noise signal was kept at 65 dB_SPL_ throughout the experiment. This approach requiring level changes of both the signal and noise tracks was preferred over designs where either the speech or noise level is kept constant, as the latter may result in very significant changes in loudness while sweeping the SNR range. 

Custom scripts were written in Matlab to analyze, model, and visualize the experimental data from the three studies. 

## 3. Results

This section presents the results on the final version of the corpus. The interim results of the first two studies are omitted for conciseness.

### 3.1. Spectrum and Phonemic Distribution

**Figure 1:**
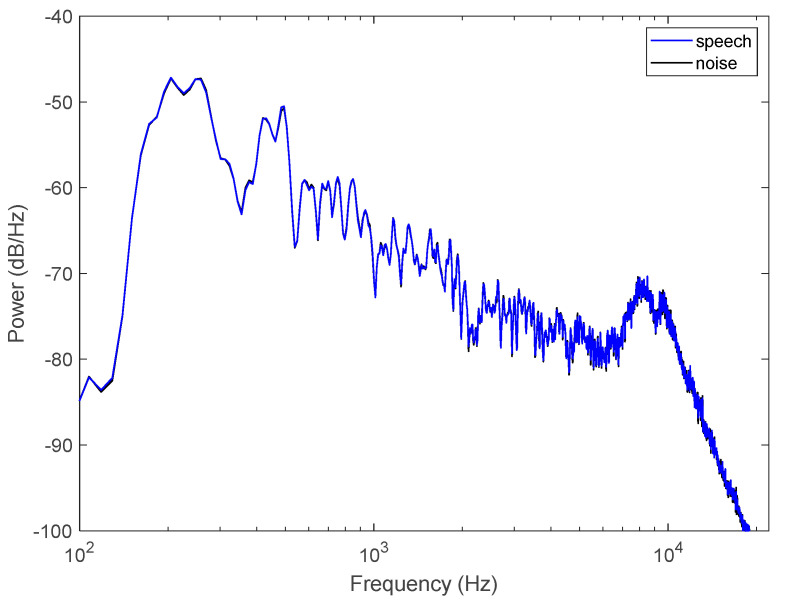
Power spectral density of the digital speech and noise signals.

The speaker is a female voice professional with standard Flemish pronunciation. To calculate the average long-term spectrum, all utterances were concatenated into a long speech file without any pauses. The Welch’s power density spectra of the clean speech, calculated with a 2048 FFT window, is shown in Figure 1. The spectrum peaks at the fundamental frequency of 202 Hz. The figure also shows the spectrum of the speech-shaped noise. By construction, they are almost indistinguishable with an average absolute difference of 0.5 dB. 

**Figure 2:**
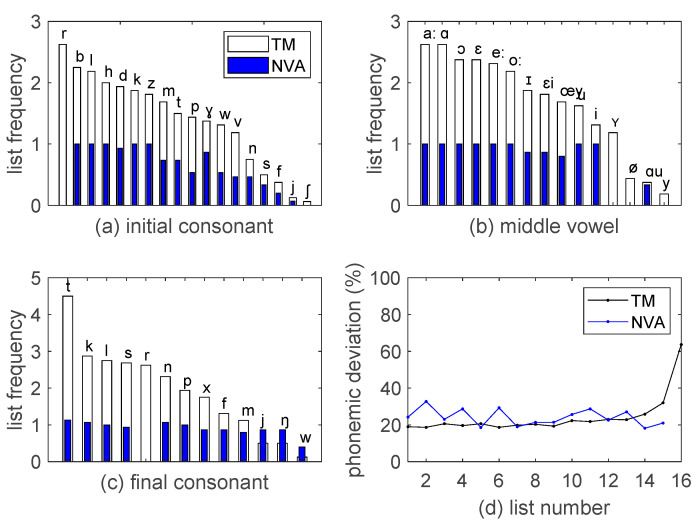
(**a**–**c**): phoneme count, averaged across lists, for initial, middle, and final position in the CVC word. (**d**): deviation from the target phonemic distribution for each list, expressed as a percentage.

The average phonemic distribution for the initial, medial, and final position, both for the Thomas More and the NVA lists, is shown in Figure 2. As an example, an average speech list of 25 words from the Thomas More corpus has 2.6 words starting with an /r/. The bottom-right panel shows the deviation from the target distribution, calculated by summing the absolute value of the difference between the actual list distribution and the target over all phonemes and normalizing this by the total number of phonemes in a list, expressed as a percentage. Most lists differ in ±15 phonemes (20%) across the 25 words from the target distribution. The last list has a higher deviation as it is composed of the remaining words in the shrunken word pool.

### 3.2. Reference Speech Recognition Curves

**Figure 3:**
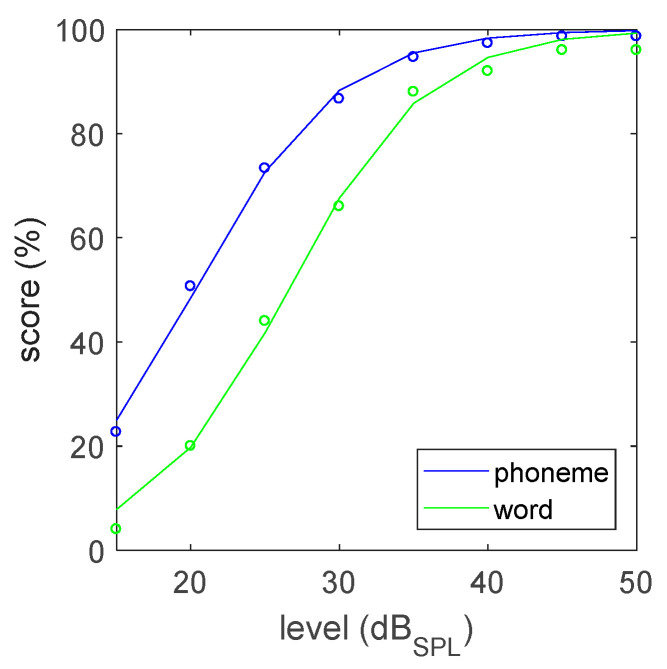
Reference speech recognition curve in quiet. Experimental data (dots) and curve fit (line).

Figure 3 shows the reference speech recognition curve for monaural listening in quiet. The curve and SRT value were obtained by first taking the median recognition score at each presentation level over all lists and over the full group of 32 participants, followed by a mathematical fit (function fminsearch in Matlab) of a sigmoid curve to the experimental data, using the formula

(1) $$y=\frac{100}{1+\exp\left(-S/25\cdot \left(x-\mathrm{SRT}\right)\right)}\;$$

whereby the x (level or SNR) and y (phoneme or word score) variables denote the experimental data and the parameters SRT and S denote the speech recognition threshold and the maximal slope (in percentage per dB).

For phoneme scoring, the threshold (SRT in quiet or SRT_q_) and slope values are respectively 20.3 ± 1.1 dB_SPL_ and 4.8 ± 0.3%/dB. For word scoring, they are respectively 26.6 ± 1.1 dB_SPL_ and 4.8 ± 0.3%/dB. The margins were calculated by fitting a sigmoid for each participant, extracting the parameters and determining the 95% confidence interval. Around the SRT level, the word score curve is well approximated by the phoneme score curve shifted by 6.3 dB_SPL_.

Perceptual list equivalence was checked for phoneme scoring at the steepest point, the SRT value. For every list and presentation level, four different participants had evaluated this condition. A list-specific speech recognition curve was obtained by taking the median value of these data points and a sigmoid fit was used to obtain the SRT estimate for each list. The 95% confidence interval estimate is 1.8 dB_SPL_. All list-specific SRT values were found to fall within the confidence interval. 

The presentation levels to reach speech recognition scores of 30–90% (10% interval) specified by the ISO standard were determined through the sigmoid curve fitting and are listed in Table 1 (left side).

**Figure 4:**
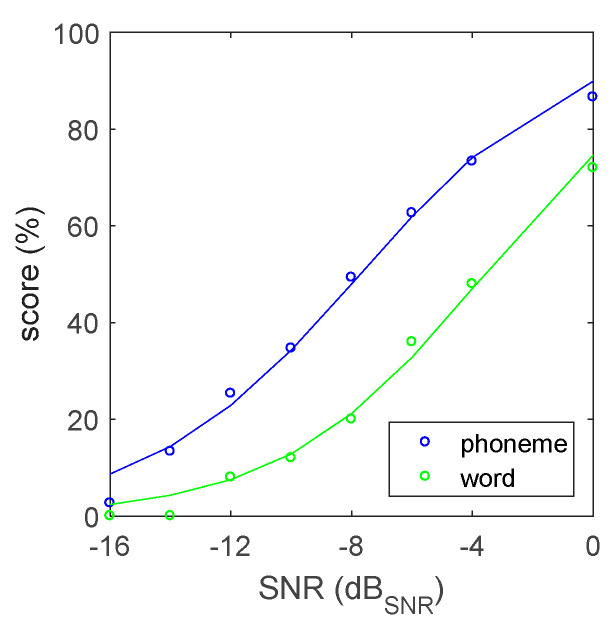
Reference speech recognition curve in speech-shaped noise. Experimental data (dots) and curve fit (line).

Similarly, Figure 4 presents the reference speech recognition score in speech-shaped noise, determined in the last study with 25 subjects. The reference speech recognition threshold level in noise (SRT_n_) for phoneme scoring is −7.7 ± 0.6 dB_SNR_ with a maximal slope of 7.1 ± 0.3%/dB. For word scoring, the threshold is −3.6 ± 0.5 dB_SNR_ with a slope of 7.5 ± 0.3%/dB. Perceptual list equivalence in noise was again checked at SRT_n_. The 95% confidence interval estimate is 1.2 dB_SNR_, with all list-specific SRT_n_ values falling within the interval. The SNR levels corresponding to the speech recognition scores of 30–90% are given in Table 1.

## 4. Discussion

This work has developed a new Flemish Dutch CVC speech audiometry corpus consisting of phonemically and perceptually balanced lists. A corpus of 16 test lists with 25 words, spoken by a female speaker, was created for clinical and research use. By design, its phoneme distribution matches the general phoneme statistics of Dutch CVC words.

The development followed the process described in the ISO 8253-3:2012 standard. For most aspects, the requirements were met. Some aspects were challenging. Towards perceptual balance of the lists, the ISO standard requires that no single test item has an individual SRT deviating by more than 3 dB from the average single item SRT across the corpus. This requirement is intended to equalize the intelligibility of all single test items, thereby maximizing the steepness of the speech recognition curve. Although steepness is a desirable property, strict adoption of this criterion would have greatly reduced (at least halved) the number of words, and consequently the number of lists the new corpus would contain. A larger corpus with more lists and better phonemic balance was a key design goal and was considered higher priority compared to steepness of the reference speech recognition curve. Therefore, a more pragmatic 5 dB criterion was used for the word selection. In the final test, a slope of 4.8%/dB was achieved for phoneme recognition in quiet, which is identical to the value reported by Wouters et al. for the Flemish NVA corpus [4].

Also, their respective SRT values in quiet are similar, respectively 20.3 (Thomas More) and 19.0 dB_SPL_ (NVA). The Flemish version of the NVA speech audiometry lists were validated in quiet with a similar number (30) of young normal hearing subjects [4].

For speech understanding in noise, a speech-shaped noise signal was created exactly matching the long-term average speech spectrum of the new materials. To determine the reference speech recognition curve, the level of the combined speech and noise signals was kept constant at 65 dB_SPL_. This approach was chosen over keeping the speech or noise level fixed to avoid significant loudness changes when testing over a wide range of SNRs. The resulting SRT_n_ and slope are −7.9 dB_SNR_ and 7.1%/dB, whereas the values for the Flemish NVA corpus are −9.3 dB_SNR_ and 5.9%/dB. These tests were taken with a fixed noise level [20].

The new speech materials can be used for various scenarios, such as testing at a fixed presentation level of SNR, or adaptive determination of the SRT in quiet or noise. In this paper, we reported on the reference speech recognition curve. The ISO norm requires the reporting of perceptual equivalence and average test-retest repeatability for every condition of interest with at least 10 ontologically normal hearing persons. Detailed validation of these scenarios is the topic of further studies.

The test was designed for use with adult listeners. No effort was made to validate the materials for young children.

Sometimes a listener undergoing testing at soft levels may realize too late that a speech list has already started. Therefore, in the final release of the CD, as in the NVA corpus, an initial training word was added to every speech list. The word was randomly chosen and should not be scored. Determination of the phoneme score is achieved by counting the correctly identified phonemes from test items 2–26, dividing by three and multiplying by four. The CD also contains the calibration signals required by the ISO standard.

Clinicians may be interested in creating more lists by splitting the lists in half. We informally explored in the post-analysis if half lists would satisfy the list equivalence criterion, but this was not the case. Use of half lists is therefore discouraged. Additionally, while it may be insightful for a clinician to look in more detail into the specific phoneme errors a person is making [21], accurate determination of the phoneme confusion matrix is not possible with a list length of 25 words.

The speech materials have been added to this paper as supplementary materials, making them available to the research and clinical communities. They are easy to use in an audiometric setting, as the same calibration settings as for the common NVA test apply. They may be a useful extension to the NVA test when CVC testing in a large number of conditions is required.

## Figures and Tables

**Table 1:** Sound levels for different speech recognition scores in quiet and in noise (phoneme and word scoring).

Speech Recognition Score %	Quiet (dB_SPL_)		In Noise (dB_SNR_)	
	Phoneme	Word	Phoneme	Word
30	16.2	22.6	−10.7	−6.4
40	18.3	24.7	−9.1	−5.0
50	20.3	26.6	−7.7	−3.6
60	22.2	28.5	−6.3	−2.2
70	24.4	30.5	−4.7	−0.8
80	27.0	33.0	−2.8	1.0
90	30.8	36.8	0.0	3.7

## Data Availability

The word materials, speech materials, and study results presented are available in the supplementary material of this article.

## Supplementary Materials

The following supporting information can be downloaded at: https://www.mdpi.com/article/10.3390/audiolres12040041/s1.

## Abbreviations

CVC	Consonant-vowel-consonant
CI	Cochlear implant
IPA	International Phonetic Alphabet
NVA	Nederlandse Vereniging Audiology (Dutch association of Audiology) lists
PAT	phoneme allocation table
SRT, SRT_q_, SRT_n_	Speech recognition threshold level. Subscript indicates in quiet or in noise
